# Differential Non-Volatile Metabolomics in High- and Low-Alcohol Strong-Flavor Baijiu by Non-Targeted Approach

**DOI:** 10.3390/foods15010037

**Published:** 2025-12-22

**Authors:** Yuxia Fan, Chenxi Qiu, Panpan Chen, Yajiao Zhao, Xiaoxiao Feng, Shui Jiang, Dengyong Liu, Yufa Cao, Shi Liu, Yuan Liu

**Affiliations:** 1 Department of Food Science & Technology, School of Agriculture & Biology, Shanghai Jiao Tong University, Shanghai 200240, China; nancyfyx@sjtu.edu.cn (Y.F.); qiuchenxi0818@163.com (C.Q.); chenpanpan0808@163.com (P.C.); zhaoyj0820@sjtu.edu.cn (Y.Z.); fxxhzl@sjtu.edu.cn (X.F.); jiangshui@sjtu.edu.cn (S.J.); 2 College of Food Science and Engineering, Bohai University, Jinzhou 121013, China; jz_dyliu@126.com; 3 Suqian Product Quality Supervision and Testing Institute, Suqian 223800, China; 4 School of Food Science and Engineering, Ningxia University, Yinchuan 750021, China

**Keywords:** strong-flavor baijiu, non-targeted metabolomics, nonvolatile compounds, multivariate analysis

## Abstract

Alcohol level is a critical quality parameter in Chinese baijiu, significantly influencing its flavor profile, sensory characteristics, and overall quality, which in turn affect consumer preferences and the development of the liquor industry. Understanding the non-volatile compounds in strong-flavor baijiu (SFB) is essential for elucidating its taste and mouthfeel characteristics. This study aims to identify non-volatile compounds using a non-targeted metabolomics approach and investigate the differences between high- and low-alcohol SFB from the Jiangsu region. A total of 647 non-volatile compounds were quantified. The key differential metabolites were screened among different samples. In total, 110 key differential non-volatile compounds were identified and quantified, which displayed significant differences between high- and low-alcohol SFB samples and showed notable similarities in compound types. Furthermore, the variation in non-volatile profiles among samples of the same brand but different alcohol levels was investigated. The different brands and origins of SFB samples were identified using OPLS-DA. The results indicated that the key non-volatile metabolites in most of the high-alcohol samples were higher than those in low-alcohol samples. This study provides valuable insights into the impact of alcohol level on the non-volatile composition of SFB, offering a theoretical foundation for flavor characterization, quality control, and standardization in baijiu production.

## 1. Introduction

Baijiu, as one of the six main distilled spirits in the world, is a traditional Chinese liquor with a distinct flavor system developed over hundreds of years in China [1,2]. Strong-flavor baijiu (SFB) is a typical and popular type of baijiu that holds over 70% of the baijiu market [3]. According to data from the National Bureau of Statistics, China’s liquor production reached 4.145 million kiloliters in 2024, with the estimated output of strong-aroma-type baijiu being approximately 2.9 million kiloliters [4]. SFB is typically produced through natural solid-state fermentation and distillation processes. Sorghum, or a combination of corn, rice, millet, glutinous rice, wheat, and other grains according to specific recipes, is the main raw material [5]. The distinctive flavor of SFB arises from the synergistic contribution of raw grains, Daqu, pit mud, a complex microbial consortium, and the site-specific environmental conditions [6,7]. SFB is characterized by its fragrant flavor, soft mouthfeel, and long aftertaste, which are largely attributed to a diverse array of volatile and nonvolatile compounds generated during fermentation, distillation, and aging [8,9]. While the composition and sensory contribution of volatile compounds in baijiu have been extensively studied [10,11], non-volatile compounds also play a critical role in shaping the overall flavor profile and sensory perception of baijiu [12]. However, research on non-volatile compounds, especially in strong-flavor baijiu from the Jiangsu region, has not yet been systematically conducted.

Non-targeted metabolomics has emerged as a powerful analytical strategy for comprehensively characterizing the complex chemical composition and underlying flavor mechanisms in food. This approach enables the global profiling of a wide range of small molecules using advanced analytical techniques such as high-resolution mass spectrometry (HRMS), ultra-high-performance liquid chromatography (UHPLC–MS), and nuclear magnetic resonance (NMR) spectroscopy. In baijiu research, non-targeted metabolomic analyses have facilitated a systematic exploration of the flavor matrix, revealing the biochemical pathways and molecular interactions underlying baijiu’s sensory characteristics. Chemical isotope labeling–LC–MS methods have identified hundreds of non-volatile compounds in different baijiu aroma types, providing potential biomarkers for classification and quality control [13,14]. Non-targeted metabolomics has also been employed to characterize the dynamic metabolic processes during baijiu fermentation [15]. Multi-timepoint analyses of fermentation substrates have revealed significant changes in phenolic metabolites, lipids, and organic acids, elucidating highly correlated metabolic pathways [16,17]. These findings underscore the significant impact of raw material selection on metabolite diversity and the resultant flavor profile, highlighting its critical role in determining the sensory characteristics of food products. Recent multi-omics approaches have integrated non-targeted metabolomics with metagenomics and flavoromics to construct a comprehensive microbiome–metabolite–flavor network [18]. This system-level analysis has revealed how microbial community dynamics drive metabolite production and, consequently, baijiu’s sensory attributes [19]. These studies demonstrate that non-targeted metabolomics has transformed baijiu research by enabling comprehensive chemical characterization, elucidating biochemical pathways of flavor formation, and offering objective tools for quality evaluation.

Traditional baijiu research has focused heavily on volatile aroma compounds, but in recent years, some studies have shifted attention toward non-volatile organic acids, amino acids, phenolics, long-chain lipids/esters, and other matrix components. Luo et al. summarized the flavor characteristics and physiological functions of four non-volatile flavor components in baijiu (non-volatile organic acids, amino acids, peptides, and polysaccharides) and reviewed the analytical methods, highlighting knowledge gaps that may guide future research directions in the baijiu flavor field [20]. Fang et al. compared typical Chinese baijiu (soy sauce aroma type liquor, strong aroma type liquor, and light aroma type liquor) and Western liquors (whisky, brandy, rum, and vodka) using metabolomics approaches [21]. The results revealed that Chinese baijiu contained higher non-volatile organic acid levels (283–727 mg/L) than Western spirits (16–378 mg/L); lactic acid was the major non-volatile acid (125–484 mg/L) in Chinese baijiu [21]. Zhang et al. applied multi-omics to investigate the change in non-volatile compounds in waxy wheat baijiu across aging years [22]. A total of 718 non-volatile metabolites (lipids, amino acids, and phenolic/organic acids) were identified. Additionally, an accumulation of physiologically active amino acids was observed during the aging process [22]. Research has demonstrated that organic acids are primarily generated during fermentation as metabolic by-products of microorganisms, such as lactic acid bacteria and yeast. The types and concentrations of organic acids are significantly influenced by fermentation conditions, including temperature, pH, and duration, as well as the biochemical composition of the raw materials [23]. Amino acids are predominantly released through the enzymatic hydrolysis of proteins in raw materials such as sorghum and rice [24,25]. Peptide components originate from both proteolytic degradation of dietary proteins and microbial metabolic activities [26,27]. Polysaccharides, primarily consisting of starch and cellulose, are hydrolyzed by microbial enzymes into low-molecular-weight sugars, which are subsequently metabolized into a range of flavor-active compounds [25,28]. Ethanol concentration not only modulates the solubility and stability of flavor compounds but also affects the perception of sweetness, bitterness, and umami through interactions with nonvolatile components [29]. However, the impact of alcohol content on the composition and distribution of nonvolatile metabolites in SFB remains insufficiently explored.

The primary objective of this study was to identify non-volatile compounds in strong-flavor baijiu (SFB) using a non-targeted metabolomics approach and to investigate the differences between high alcohol and low alcohol SFB samples. To achieve this, we systematically analyzed 12 pairs of SFB samples from the Jiangsu region using ultra-high-performance liquid chromatography coupled with a Q Exactive Plus mass spectrometer (UHPLC-Q Exactive Plus MS). Key differential metabolites among the different samples were screened and compared to elucidate the differences between high-alcohol and low-alcohol samples. Furthermore, orthogonal partial least squares (OPLS-DA) was used to establish models to distinguish the brands and origins of SFB. The findings of this research were expected to contribute to quality control and product standardization in baijiu production.

## 2. Materials and Methods

### 2.1. Samples

In this study, a total of 12 pairs of strong-flavor baijiu samples were collected in Jiangsu province, China. Each pair consisted of one high-alcohol sample and one low-alcohol sample, and these samples included different origins and brands. The detailed information about all samples is shown in Table 1. All samples were stored at room temperature and in a dry environment for further analysis.

### 2.2. Chemicals and Materials

HPLC-grade ethanol (>99.0%) was purchased from ANPEL Laboratory Technologies Inc. (Shanghai, China). The internal standards used were 2-chlorophenylalanine (≥99.0%, CAS:14091-11-3) and ethyl cinnamate (≥99.0%, CAS:103-36-6), which were supplied by Macklin Biochemical Co., Ltd. (Shanghai, China). The standard solution was prepared by dilution with ultrapure water.

### 2.3. UHPLC-Q Exactive Plus Mass Analysis

#### 2.3.1. Sample Preparation

Each sample (3 mL) was spiked with 2-chlorophenylalanine and ethyl cinnamate (both at 0.5 μg/mL) as internal standards for subsequent quantitative analysis. All samples with internal standards were centrifuged at 10,000 rpm for 15 min at 24 °C to remove potential impurities (Centrifuge 3K15 refrigerated centrifuge, Sigma-Aldrich, Darmstadt, Germany), and the supernatants were then transferred to a 2 mL vial for a non-targeted metabolomic analysis. Quality control (QC) samples were obtained by mixing 20 μL of each test sample. Each sample was analyzed in triplicate.

#### 2.3.2. Chromatographic Conditions

An ultra-high-performance liquid chromatography (UHPLC) system coupled with a Q Exactive Plus mass spectrometer (QE-MS) (Thermo Fisher Scientific, Waltham, MA, USA) was employed for the analysis of the baijiu samples. Chromatographic separation was performed on an ACQUITY UPLC HSS T3 column (100 mm × 2.1 mm, 1.7 μm, Waters, Milford, MA, USA) at a column temperature of 45 °C. The sample was injected at a volume of 1 μL using an autosampler. The mobile phases consisted of (A) water containing 0.1% formic acid (*v*/*v*) and (B) methanol containing 0.1% formic acid (*v*/*v*). The gradient elution program was as follows: the proportion of mobile phase A was decreased from 99% to 0% over 12 min, while mobile phase B was increased from 1% to 100% within the same period. The column was then equilibrated at 0% A/100% B for an additional 12 min. The flow rate was maintained at 0.4 mL/min.

#### 2.3.3. Mass Spectrometry Conditions

The Q-Exactive Plus Orbitrap MS (Thermo Scientific, Waltham, MA, USA) was equipped with a heated electrospray ionization source (HESI). Each sample was subjected to electrospray ionization (ESI) in positive and negative ion modes, respectively. The target compounds were analyzed in data-dependent mode (DDA) with one full scan followed by the top 10 MS/MS scans. Collision energy was NEC 15 and 30 to fragment the ions. Nitrogen (99.999%) was used as the collision-induced dissociation gas. The full scan resolution was 70,000 with an AGC target of 5.00 × 10^5^ and a maximum injection time of 50 ms. The full scan range was 67–1000 amu, the spray voltage was set to 3.2 kV (positive mode) and 2.8 kV (negative mode), and the capillary temperature was held at 320 °C. The S-lens RF level was set to 50 V.

### 2.4. Multivariate Statistical Analysis and Network Visualization

Metabolite identification was performed based on a mass error tolerance of ±3 ppm, isotope pattern similarity > 75%, and accurate mass matching to reference databases. To ensure reliable identification, MS/MS spectra were acquired for all detected ions, and the resulting MS/MS data were retained for further analysis. The internal standard method was used for the quantitative analysis, and the mass concentration of each compound was calculated based on the concentration of the internal standard (0.5 μg/mL). All data were presented as mean ± standard deviation (SD) to ensure stability and reliability. A free online data analysis platform (Metware Cloud, https://cloud.metware.cn/) was used for heatmap and volcano plot analysis. A supervised PLS-DA was built in SIMCA 14.0 (Umetrics, Umea, Sweden). The Hotelling’s T^2^ ellipse drawn on every score plot delimits the 95% confidence boundary of the modeled variability. Overfitting was diagnosed with 200 random-permutation cross-validations, and compounds whose variable-importance-in-projection (VIP) exceeded 1.0 were retained as candidate markers. Significance levels of metabolites between intergroups were calculated by one-way ANOVA at *p* < 0.05 with Dunnett’s multiple comparisons test using SPSS 22.0 software (version 29.0, Chicago, IL, USA).

## 3. Results and Discussion

### 3.1. Analysis of Non-Volatile Metabolic Compounds in SFB

**Figure 1:**
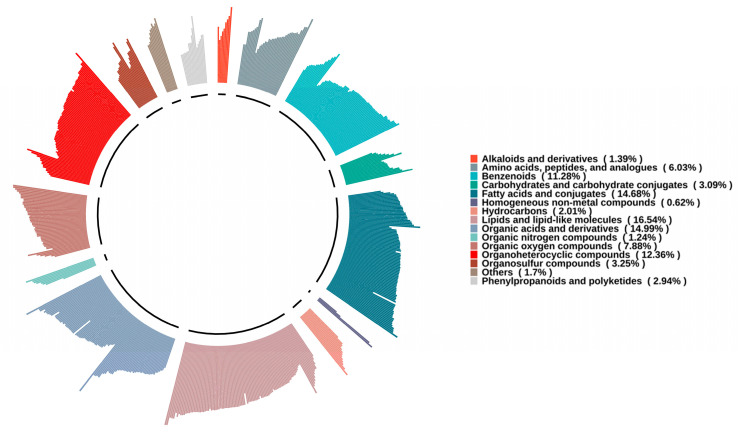
Classification circular diagram of non-volatile metabolic compounds in high- and low-alcohol SFB.

Non-targeted metabolomics analysis was applied to investigate the differences in high- and low-alcohol SFB metabolites via UHPLC-Q Exactive Plus mass spectrometry. A total of 647 non-volatile metabolites were identified, comprising 473 compounds in positive ion mode and 174 compounds in negative ion mode, all of which were matched to HMDB. Figure 1 presents the classification of all compounds.

The results demonstrated that these non-volatile compounds were assigned to 15 categories, including lipids and lipid-like molecules (107), organic acids and derivatives (97), fatty acids and conjugates (95), organoheterocyclic compounds (80), benzenoids (73), organic oxygen compounds (51), amino acids, peptides, and analogs (39), organosulfur compounds (21), carbohydrates and carbohydrate conjugates (20), phenylpropanoids and polyketides (19), hydrocarbons (13), alkaloids and derivatives (9), organic nitrogen compounds (8), homogeneous non-metal compounds (4), and others (11). All compound information is provided in Table S1. Notably, lipids and lipid-like molecules made up the highest proportion, accounting for 16.36% of all non-volatile compounds. L-Lactic acid was the organic acid with the highest content across all the samples, indicating that L-lactic acid was one of the important non-volatile organic acids in SFB, which is consistent with previous research findings [30,31]. While no differences were observed in the types of non-volatile compounds between high- and low-alcohol samples, significant variations in metabolite content and abundance were evident among the samples.

**Figure 2:**
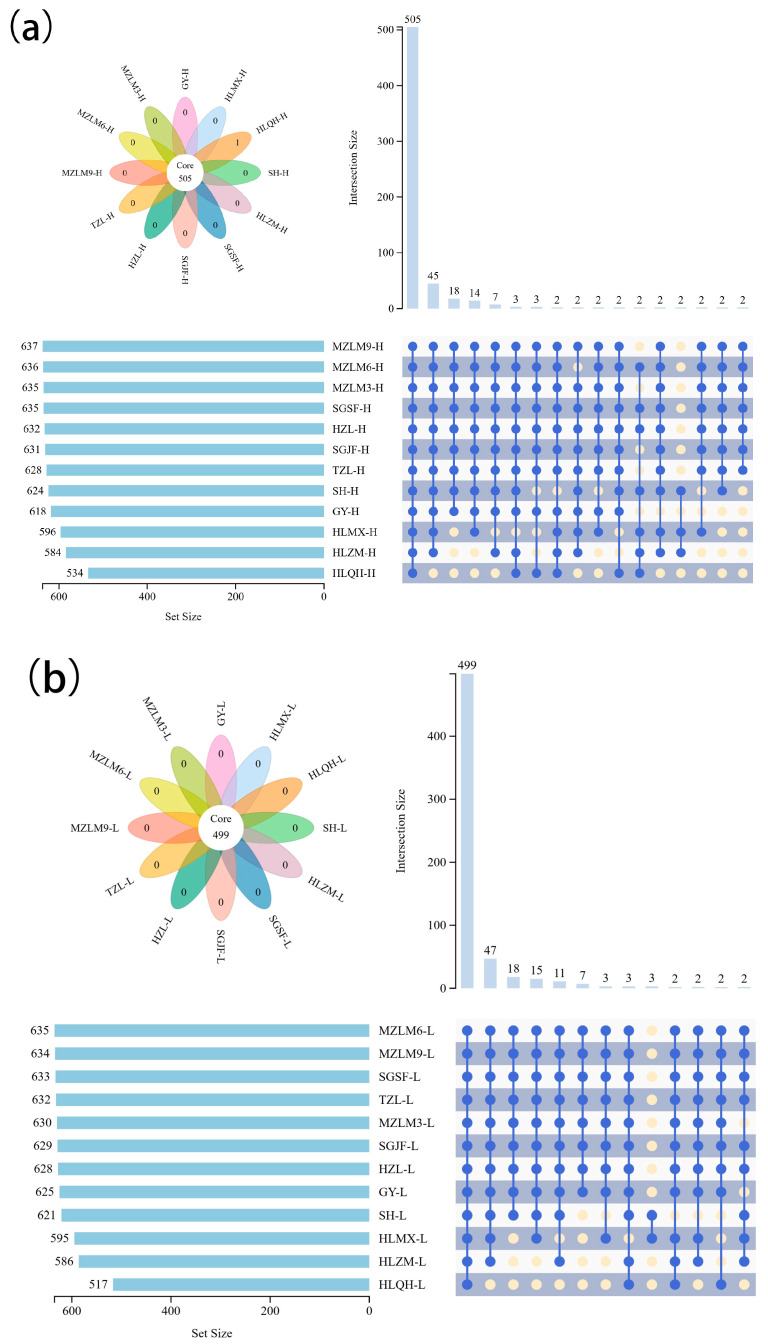
Upset plot embedded Venn diagrams comparing the non-volatile metabolites for high (**a**) and low (**b**) alcohol SFB. The horizontal bars represent the total number of non-volatile metabolites for each sample; the vertical bars or intersections represent the number of non-volatile metabolites that were shared by different samples. Where a blue filled circle is placed in the corresponding matrix cell to indicate part of an intersection, a light yellow circle is shown when not involved in the intersection. For higher visual clarity, an intersection size cut-off of <2 was introduced.

To better understand the variation in non-volatile metabolite composition between high- and low-alcohol SFB samples, an advanced Upset plot was generated based on the metabolite data (Figure 2). The plot displayed the degree of overlap and unique metabolite subsets across the different sample groups. The analysis revealed that 505 and 499 metabolic signals were present in all high-alcohol and low-alcohol samples, respectively. Statistical analysis of shared metabolites demonstrated a substantial overlap in non-volatile compounds (Venn diagrams in Figure 2). These findings indicated a high degree of similarity in the non-volatile metabolite profiles of SFB produced in Jiangsu, particularly among samples with similar alcohol content, which is a predictable outcome based on the sample setup. However, non-volatile substances play a crucial role in regulating flavor, taste, and overall flavor balance of baijiu [32,33]. Thus, an in-depth investigation into the differences in non-volatile compounds among samples with different alcohol levels and brands is essential for elucidating the formation mechanisms of baijiu flavor characteristics.

### 3.2. Screening of Non-Volatile Differential Metabolites Between High- and Low-Alcohol SFB

**Figure 3:**
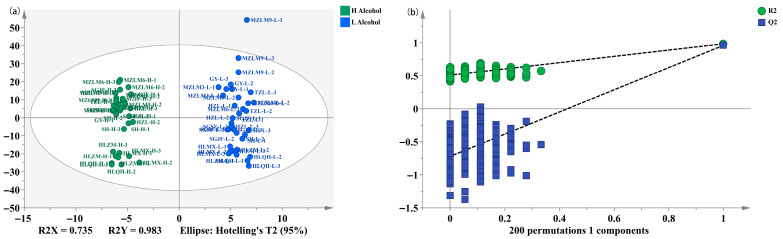
OPLS-DA of score scatter plots (**a**) and permutation test (**b**).

UHPLC-Q Exactive Plus mass qualitative analysis indicated that the types of non-volatile compounds in SFB were largely similar, but their relative concentrations varied significantly. In order to better visualize the differences and further clarify the differential metabolites, orthogonal partial least squares discriminant analysis (OPLS-DA) was performed based on non-volatile compounds, which is considered a very effective method for sample classification [34] and extensively employed in metabolomics analysis for analyzing the relationship between metabolites and sample groups [35]. The OPLS-DA score plot is shown in Figure 3a. The data points were clearly separated for the high- and low-alcohol SFB samples, indicating that there were significant differences in non-volatile compounds for these two groups of samples. To further test the effectiveness of this OPLS-DA model, a permutation test (200 times) was carried out, and the result is shown in Figure 3b. The parameters of the model R^2^X, R^2^Y, and Q^2^ were 0.735, 0.983, and 0.959, respectively. The regression line of Q^2^ intersected the vertical axis with a negative value at the intersection point. As is well known, R^2^X, R^2^Y, and Q^2^ are the parameters to evaluate the goodness of fit, reliability, and predictive ability of the model, respectively [36,37]. The results confirmed that a robust and reliable OPLS-DA model was obtained, with no signs of overfitting and high predictive accuracy.

**Figure 4:**
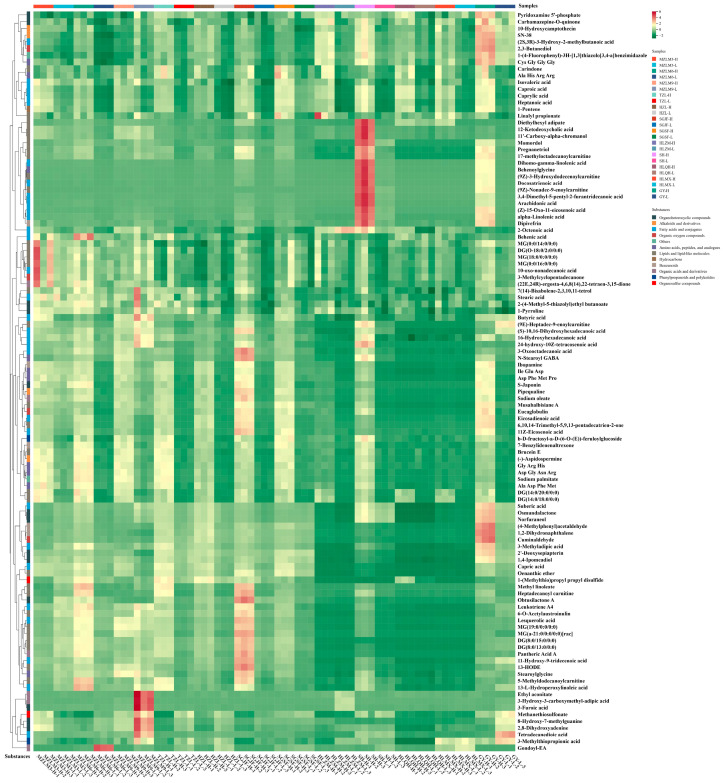
Heatmap analysis of critical metabolites in 24 SFB samples. Each column represents a baijiu sample, and each row represents a critical metabolite. A color-coded scale grading from green to red refers to the content of critical metabolite shifting from low to high.

In addition, OPLS-DA was combined with variable importance in projection (VIP) analysis to screen the most important non-volatile metabolites for all high- and low-alcohol SFB samples. The selection for different metabolites was based on their statistical significance according to the following criteria: VIP  >  1.0 from OPLS-DA analysis and *p*  <  0.05, which were considered significant for high- and low-alcohol SFB samples. A total of 110 compounds (VIP > 1, *p* < 0.05) were selected, including 32 lipids and lipid-like molecules, 7 organic acids and derivatives, 32 fatty acids and conjugates, 12 organoheterocyclic compounds, 5 benzenoids, 2 organic oxygen compounds, 10 amino acids, peptides, and analogs, 2 organosulfur compounds, 1 phenylpropanoid or polyketide, 1 hydrocarbon, 4 alkaloids and derivatives, and 2 others. Among these, lipids and lipid-like molecules, fatty acids and conjugates, organoheterocyclic compounds, amino acids, peptides, and analogs were the top four categories in terms of metabolite numbers. Detailed information on these critical metabolites is shown in Table 2 and Table S2. The samples were sourced from 12 different brands of commercial baijiu, with two alcohol levels for each brand, and the inherent diversity among the samples complicated the identification of universally consistent trends. Overall, the key differential metabolites were generally more abundant in most high-alcohol samples compared to their low-alcohol counterparts. Moreover, the high-quality samples based on price evaluation exhibited higher concentrations and greater diversity of key non-volatile compounds. As shown in Figure 4, a heatmap visualized the relative abundances of these 111 critical metabolites. The observed differences in key nonvolatile metabolites are not only related to alcohol content but also closely associated with these key factors, such as raw materials, fermentation progress, and blending techniques [38].

The raw materials (e.g., sorghum, rice, wheat, and barley) are a fundamental determinant of the metabolic landscape in baijiu production. Different grains possess distinct starch compositions, protein content, and microelement profiles, which influence the availability of carbon and nitrogen sources for microbial metabolism during fermentation [39]. For instance, sorghum, the primary grain in strong-flavor baijiu, contains higher levels of polyphenols and tannins, which can be transformed into phenolic acids and other nonvolatile compounds via microbial action [16]. This transformation directly affects the final flavor profile and mouthfeel of the liquor. Fermentation progress, particularly the duration, temperature, and microbial community dynamics, plays a crucial role in modulating nonvolatile metabolite accumulation. Studies have demonstrated that extended fermentation cycles promote the biosynthesis of organic acids (e.g., lactic acid, acetic acid), amino acids, and polyols, which contribute to the complexity and fullness of the liquor [40]. Moreover, the dominance of specific microbial species (e.g., *Lactobacillus*, *Bacillus*, and *Saccharomyces*) during different fermentation phases can lead to the differential production of nonvolatile metabolites such as nucleotides, peptides, and polysaccharides, which are known to enhance the umami taste and lingering aftertaste [41]. Blending is a critical process in baijiu production that enables the harmonization of different fermented liquors, including base liquor, aged liquor, and special flavor components. These components exhibit significant variations in their metabolite profiles due to differences in fermentation duration, microbial consortia, and aging conditions. Through controlled blending, not only is the alcohol content standardized, but also a synergistic integration of nonvolatile compounds, such as esters, organic acids, and polyphenols, is achieved, resulting in a more complex and balanced sensory profile [42]. For instance, the incorporation of aged liquor, which is enriched in advanced Maillard reaction products and melanoidins, significantly enhances the liquor’s body, mouthfeel, and aftertaste, contributing to its overall sensory maturity and complexity [43]. In this study, considering the sample settings, these differences in nonvolatile metabolites might be mainly due to the influence of these factors, which lead to variations in the taste, full-bodied mouthfeel, and aftertaste characteristics of different strong-flavor baijiu.

### 3.3. Comparison and Differential Metabolic Analysis of High- and Low-Alcohol SFB

**Figure 5:**
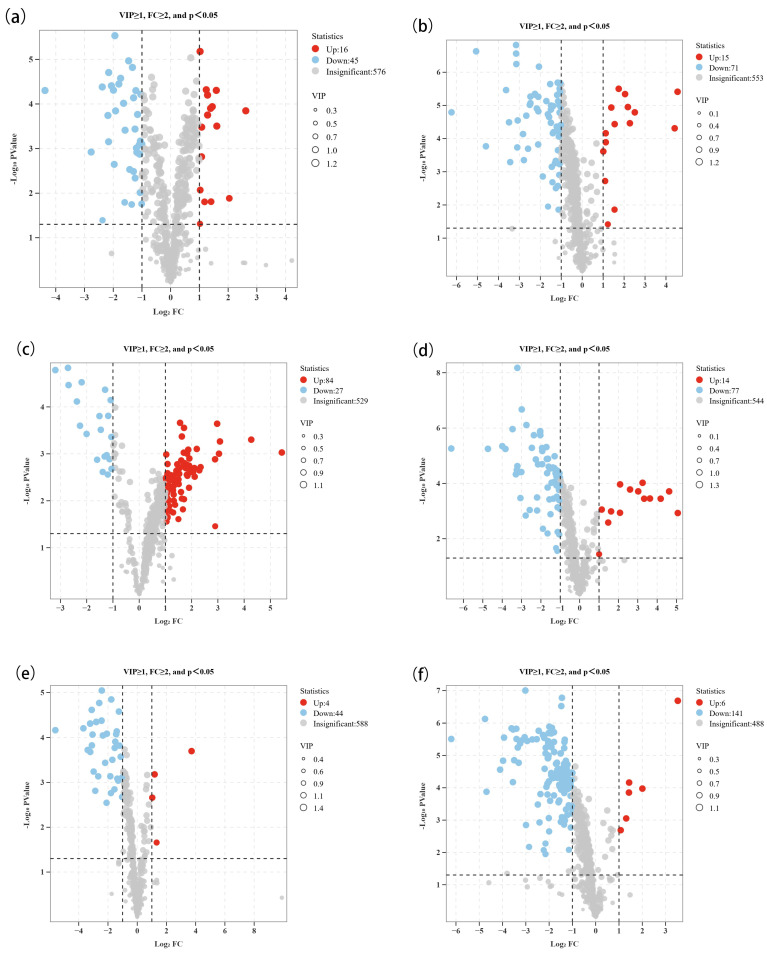

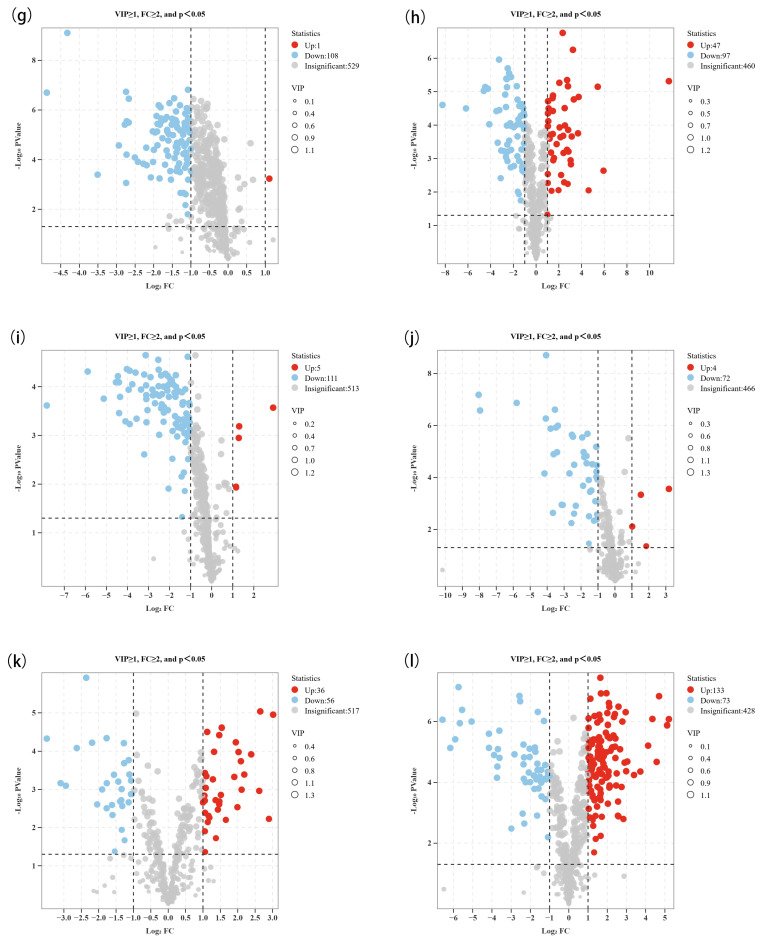
Volcano plots of metabolites for 12 pairs of SFB, including MZLM3 (**a**), MZLM6 (**b**), MZLM9 (**c**), TZL (**d**), HZL (**e**), SGJF (**f**), SGSF (**g**), HLZM (**h**), SH (**i**), HLQH (**j**), HLMX (**k**), and GY (**l**); Each point in the volcano diagram represents a metabolite, and the size of the scatter point represents the VIP value, where blue points represent down regulated differential metabolites, red points represent up regulated differential metabolites, and gray points represent detected metabolites with insignificant differences.

To further compare the differences in non-volatile compounds among samples with varying alcohol levels within the same sample set, the differential metabolite analysis was conducted for each of the 12 sample groups. Volcano plots were generated to highlight significantly altered metabolites based on the following criteria: VIP > 1, fold change (FC) > 2.0, and *p* < 0.05 (Figure 5). These plots provided a refined visualization of the number of up-regulated and down-regulated metabolites in each sample group. For instance, Figure 5a shows that 16 metabolites were up-regulated and 45 were down-regulated between high- and low-alcohol MZLM3 samples. Similarly, the differential compound analysis results for the other samples are shown in Table 3. Compared to the high-alcohol samples, low-alcohol samples generally exhibited fewer up-regulated and more down-regulated key differential metabolites, with the exceptions of MZLM9 (Figure 5c) and GY (Figure 5l). The alcohol content significantly influences flavor characteristics, yet it is not the decisive factor in determining the quantity of non-volatile compounds [44]. The changes in non-volatile metabolites of each pair of samples indicated that most of the high-alcohol SFB contained higher levels of key non-volatile compounds, contributing to the differences in flavor characteristics between high- and low-alcohol SFB.

Studies of the influence of alcohol content on both volatile and non-volatile compounds in alcoholic beverages support the findings of the present research. It was observed that higher alcohol content led to a significant increase in both volatile and non-volatile compounds in the optimization of fermentation processes for lychee wine. A total of 544 key differential metabolites were identified before and after fermentation, with 334 up-regulated and 210 down-regulated. This finding underscores the role of alcohol content in promoting the accumulation of non-volatile compounds, thereby enhancing flavor profiles [45]. In wine research, it has been demonstrated that changes in alcohol concentration significantly impacted the sensory attributes of wine, particularly taste (sweetness, acidity) and palate. Within the range of 12–15% (*v*/*v*) alcohol content, red wines exhibited altered astringency, while white wines showed variations in bitterness, which indicated that the alcohol content can alter the overall flavor of wine by affecting the content and perception of both volatile and non-volatile compounds [46]. Analysis of non-volatile compounds in gin with different alcohol contents revealed that phenolic compounds, organic acids, and other compounds exhibited different content distributions, which were similar to the changes in non-volatile metabolites observed in this study [47]. Therefore, high alcohol content can contribute to dissolving aromatic substances such as esters and acids, allowing them to dissolve more fully into the liquor and enrich its flavor. Conversely, low alcohol content may reduce the solubility of certain non-volatile compounds, affecting flavor release.

### 3.4. Discrimination of the Different Categories of Strong-Flavor Baijiu

**Figure 6:**
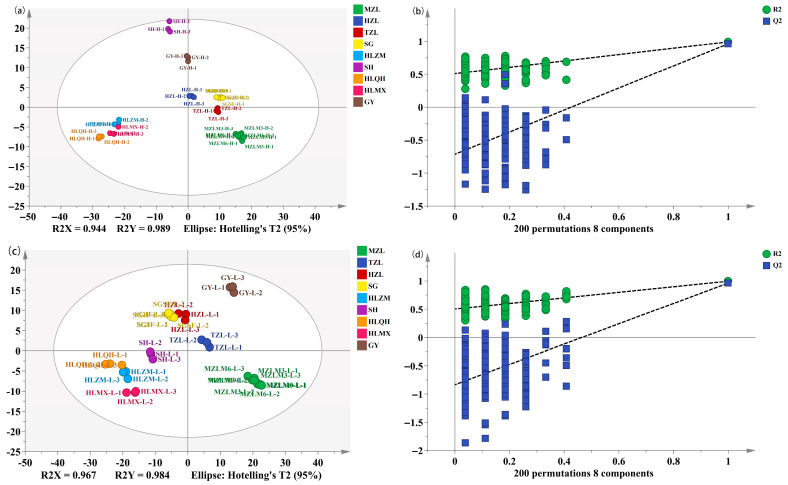
Discrimination of the different brands of strong-flavor baijiu of high- or low-alcohol level by OPLS-DA. Score scatter plots (**a**) and permutation test (**b**) for high-level samples; score scatter plots (**c**) and permutation test (**d**) for low-level samples.

Furthermore, discriminant analysis was performed on the different categories of SFB with the same alcohol content by OPLS-DA. The samples were classified based on origin and brand. The origins were classified into three categories, namely YHSQ, SHSQ, and LSHA (Table S3). The brands were categorized into nine classes, which were MZL, TZL, HZL, SG, HLZM, SH, HLQH, HLMX, and GY (Table S3). The OPLS-DA models were established for high alcohol samples and low alcohol samples, respectively. Figure 6 shows the results of the discriminant analysis between different brands of SFB. The parameters of R^2^X, R^2^Y, and Q^2^ correlated with different brands of high alcohol SFB were 0.944, 0.989, and 0.935 for high alcohol SFB, respectively (Figure 6a); the corresponding values were 0.967, 0.984, and 0.895 for low alcohol SFB (Figure 6c). These values suggested that the OPLS-DA models were stable and reliable. In the OPLS-DA plot, the separation between brand groups was more distinct for high-alcohol samples compared to low-alcohol samples, indicating that the brand-specific differences were more pronounced in high alcohol SFB samples.

**Figure 7:**
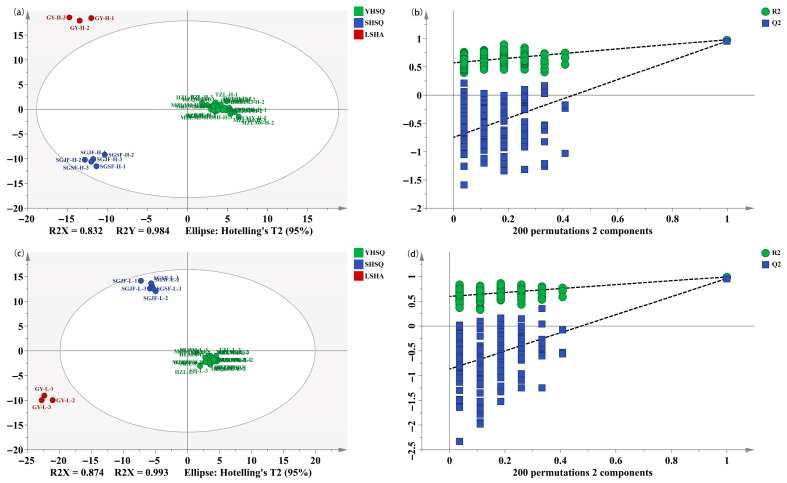
Discrimination of the different origins of strong-flavor baijiu of high or low alcohol level by OPLS-DA; score scatter plots (**a**) and permutation test (**b**) for high level samples, and score scatter plots (**c**) and permutation test (**d**) for low level samples.

To further validate and evaluate the performance of the OPLS-DA model, permutation tests (n = 200) were conducted to verify the effectiveness of OPLS-DA. The permutation tests produced R^2^ = (0, 0.509) and Q^2^ = (0, −0.713) for high alcohol samples (Figure 6b), and R^2^ = (0, 0.504) and Q^2^ = (0, −0.834) for low alcohol samples (Figure 6d), which confirmed the high performance and effectiveness of OPLS-DA. Similarly, the results of the OPLS-DA models for identifying the different origins of SFB, shown in Figure 7. The SFB samples from the three origins were distinctly separated, indicating that differences in sample flavor characteristics lead to clear regional product attribute differences, which is of great significance for the formation of unique product styles. OPLS-DA is a common multivariate analysis method frequently employed in food research. Luo et al. constructed an OPLS-DA model to discriminate the brand and geographical origin of sauce-flavor baijiu, which showed excellent evaluation parameters [48]. He et al. used SPME-MS to detect 65 baijiu samples of six different types and used OPLS-DA for origin classification. The results showed good discriminative ability [49]. Zhang et al. established a PLS-DA model to distinguish the origin (Guizhou, Sichuan, and other regions) of Jiangxiang-type baijiu (JXB) by using 34 selected key compounds; the results showed that the model can effectively distinguish JXB [50]. In addition, the application of different machine learning methods provides a new technological approach for evaluating its flavor quality [51,52]. These methods are utilized to handle intricate omics data, enabling the analysis of distinctions and relationships between samples.

## 4. Conclusions

In this study, a non-targeted metabolomics approach was employed to identify the differences in non-volatile compounds among strong-flavor baijiu samples with varying alcohol levels. Based on the OPLS-DA model and VIP value screening, a total of 110 key non-volatile compounds displayed significant differences between high- and low-alcohol SFB samples, exhibiting notable similarities in chemical types. The differences in non-volatile metabolites across samples of the same brand but with different alcohol levels were further analyzed, and the results indicated that the key non-volatile metabolites in most high-alcohol samples were higher than those in low-alcohol samples. This differential accumulation may influence flavor profiles and contribute to the distinct sensory characteristics of SFB. Additionally, metabolite profile analysis was conducted to explore variations among samples from different brands and geographical origins. Focusing on SFB from the Jiangsu region, this study provides insights into the impact of alcohol level on non-volatile composition, offering a theoretical basis for understanding flavor variation, enhancing quality control, and promoting product standardization in baijiu production.

## Figures and Tables

**Table 1:** Detailed information about the 24 SFB samples from the Jiangsu region.

No.	Name	Alcohol Content (%vol)	Origin	No.	Name	Alcohol Content (%vol)	Origin
1	MZLM3-H	52	Yanghe, Suqian	2	MZLM3-L	45	Yanghe, Suqian
3	MZLM6-H	52	Yanghe, Suqian	4	MZLM6-L	40.8	Yanghe, Suqian
5	MZLM9-H	52	Yanghe, Suqian	6	MZLM9-L	45	Yanghe, Suqian
7	TZL-H	52	Yanghe, Suqian	8	TZL-L	42	Yanghe, Suqian
9	HZL-H	52	Yanghe, Suqian	10	HZL-L	42	Yanghe, Suqian
11	SGJF-H	52	Sihong, Suqian	12	SGJF-L	41.8	Sihong, Suqian
13	SGSF-H	52	Sihong, Suqian	14	SGSF-L	42	Sihong, Suqian
15	HLZM-H	52	Yanghe, Suqian	16	HLZM-L	42	Yanghe, Suqian
17	SH-H	50.8	Yanghe, Suqian	18	SH-L	40.8	Yanghe, Suqian
19	HLQH-H	52	Yanghe, Suqian	20	HLQH-L	42	Yanghe, Suqian
21	HLMX-H	52	Yanghe, Suqian	22	HLMX-L	42	Yanghe, Suqian
23	GY-H	52	Lianshui, Huai’an	24	GY-L	42	Lianshui, Huai’an

**Table 2:** Critical metabolites (VIP > 1, *p* < 0.05) responsible for the metabolomics variation between high- and low-alcohol SFB.

Class	Number	Component Name	CAS	Formula	Observed *m*/*z*	R_t_
Lipids and lipid-like molecules	1	Musabalbisiane A	143183-61-3	C_23_H_28_O_12_	495.15	7.18
	2	6,10,14-Trimethyl-5,9,13-pentadecatrien-2-one	762-29-8	C_18_H_30_O	263.24	7.99
	3	Pregnanetriol	27178-64-9	C_21_H_36_O_3_	337.27	7.28
	4	(9Z)-Nonadec-9-enoylcarnitine		C_26_H_49_NO_4_	440.37	7.16
	5	Oenanthic ether	106-30-9	C_9_H_18_O_2_	159.14	6.36
	6	MG (0:0/14:0/0:0)	3443-83-2	C_17_H_34_O_4_	325.23	6.75
	7	Diethylhexyl adipate	103-23-1	C_22_H_42_O_4_	371.32	7.68
	8	Momordol	189156-42-1	C_26_H_48_O_5_	458.38	6.93
	9	Brucein E		C_20_H_28_O_9_	413.18	7.36
	10	Linalyl propionate	144-39-8	C_13_H_22_O_2_	228.2	7.22
	11	DG(O-18:0/2:0/0:0)		C_23_H_46_O_4_	404.37	7.44
	12	17-methyloctadecanoylcarnitine		C_26_H_51_NO_4_	442.39	7.28
	13	MG(a-21:0/0:0/0:0) [rac]		C_24_H_48_O_4_	418.39	7.11
	14	(9Z)-3-Hydroxydodecenoylcarnitine		C_19_H_35_NO_5_	375.29	7.62
	15	5-Methyldodecanoylcarnitine		C_20_H_39_NO_4_	358.29	6.24
	16	(9E)-Heptadec-9-enoylcarnitine		C_24_H_45_NO_4_	412.34	7.24
	17	MG (19:0/0:0/0:0)		C_22_H44O_4_	390.36	6.67
	18	DG (8:0/13:0/0:0)		C_24_H_46_O_5_	437.32	6.83
	19	12-Ketodeoxycholic acid	5130-29-0	C_24_H_38_O_4_	391.28	7.64
	20	DG (14:0/20:0/0:0)		C_37_H_72_O_5_	619.53	13.27
	21	DG (14:0/18:0/0:0)		C_35_H_68_O_5_	591.5	11.71
	22	6-O-Acetylaustroinulin	75207-46-4	C_22_H_36_O_4_	382.29	6.69
	23	Heptadecanoyl carnitine	106182-29-0	C_24_H_47_NO_4_	414.36	7.05
	24	11′-Carboxy-alpha-chromanol		C_26_H_42_O_4_	436.34	7.64
	25	7(14)-Bisabolene-2,3,10,11-tetrol	122470-42-2	C_15_H_28_O_4_	290.23	6.28
	26	Carindone	38045-62-4	C_31_H_44_O_6_	530.35	6.65
	27	DG (8:0/15:0/0:0)		C_26_H_50_O_5_	460.4	7.05
	28	MG (18:0/0:0/0:0)	22610-61-3	C_21_H_42_O_4_	381.3	7.44
	29	MG (0:0/16:0/0:0)	23470-00-0	C_19_H_38_O_4_	353.27	7.07
	30	S-Japonin	36031-35-3	C_19_H_28_O_3_S	381.17	7.46
	31	Eucaglobulin	241130-84-7	C_23_H_30_O_12_	497.17	7.46
	32	Cuminaldehyde	122-03-2	C_10_H_12_O	149.1	6.2
Organic acid and derivatives	33	Sodium oleate		C_18_H_33_NaO_2_	349.24	7.46
	34	Ethyl aconitate	1321-30-8	C_8_H_10_O_6_	203.06	4.61
	35	3-Hydroxy-3-carboxymethyl-adipic acid		C_8_H_12_O_7_	243.05	4.61
	36	Gondoyl-EA		C_22_H_43_NO_2_	371.36	6.85
	37	Pantheric Acid A		C_22_H_40_O_3_	353.3	6.97
	38	(22E,24R)-ergosta-4,6,8(14),22-tetraen-3,15-dione		C_28_H_38_O_2_	407.29	7.44
	39	(9Z,11E,13S)-13-hydroxyoctadeca-9,11-dienoic acid	29623-28-7	C_18_H_32_O_3_	297.24	6.57
Fatty acids and conjugates	40	3-Methylthiopropionic acid	646-01-5	C_4_H_8_O_2_S	165.02	0.98
	41	Tetradecanedioic acid	821-38-5	C_14_H_26_O_4_	303.18	5.68
	42	16-Hydroxyhexadecanoic acid	506-13-8	C_16_H_32_O_3_	271.23	6.93
	43	Methyl linoleate	112-63-0	C_19_H_34_O_2_	339.25	6.60
	44	(Z)-15-Oxo-11-eicosenoic acid	182145-55-7	C_20_H_36_O_3_	323.26	6.97
	45	13 L-Hydroperoxylinoleic acid	33964-75-9	C_18_H_32_O_4_	311.22	6.24
	46	Stearic acid	57-11-4	C_18_H_36_O_2_	283.26	7.87
	47	Docosatrienoic acid	28845-86-5	C_22_H_38_O_2_	352.32	7.62
	48	11Z-Eicosenoic acid	5561-99-9	C_20_H_38_O_2_	311.29	8.42
	49	3-Methyladipic acid	3058-01-3	C_7_H_12_O_4_	183.06	5.44
	50	Butyric acid	107-92-6	C_4_H_8_O_2_	89.06	5.79
	51	Leukotriene A4	72059-45-1	C_20_H_30_O_3_	319.23	7.05
	52	24-hydroxy-10Z-tetracosenoic acid		C_24_H_46_O_3_	400.38	7.87
	53	3-Oxooctadecanoic acid		C_18_H_34_O_3_	299.26	6.83
	54	Arachidonic acid	506-32-1	C_20_H_32_O_2_	305.25	7.38
	55	Behenic acid	112-85-6	C_22_H_44_O_2_	358.37	6.24
	56	3,4-Dimethyl-5-pentyl-2-furantridecanoic acid	57818-43-6	C_24_H_42_O_3_	396.35	7.38
	57	Capric acid	334-48-5	C_10_H_20_O_2_	173.15	6.52
	58	(S)-10,16-Dihydroxyhexadecanoic acid	69232-67-3	C_16_H_32_O_4_	306.26	6.34
	59	Isovaleric acid	503-74-2	C_5_H_10_O_2_	103.08	6.16
	60	Lesquerolic acid	4103-20-2	C_20_H_38_O_3_	327.29	6.67
	61	11-Hydroxy-9-tridecenoic acid	105798-56-9	C_13_H_24_O_3_	229.18	6.48
	62	alpha-Linolenic acid	463-40-1	C_18_H_30_O_2_	279.23	6.97
	63	2-Octenoic acid	1577-96-4	C_8_H_14_O_2_	143.11	5.48
	64	Caproic acid	142-62-1	C_6_H_12_O_2_	117.09	6.2
	65	Suberic acid	505-48-6	C_8_H_14_O_4_	175.1	5.59
	66	Eicosadienoic acid	5598-38-9	C_20_H_36_O_2_	309.28	7.99
	67	10-oxo-nonadecanoic acid	820-42-8	C_19_H_36_O_3_	313.27	7.07
	68	Dihomo-gamma-linolenic acid	1783-84-2	C_20_H_34_O_2_	307.26	7.62
	69	Heptanoic acid	111-14-8	C_7_H_14_O_2_	131.11	6.2
	70	(2S,3R)-3-Hydroxy-2-methylbutanoic acid	71526-30-2	C_5_H_10_O_3_	141.05	4.77
	71	Caprylic acid	124-07-2	C_8_H_16_O_2_	145.12	6.2
Organoheterocyclic compounds	72	3-Furoic acid	488-93-7	C_5_H_4_O_3_	111.01	4.62
	73	1-Pyrroline	5724-81-2	C_4_H_7_N	70.07	1.11
	74	2-(4-Methyl-5-thiazolyl) ethyl butanoate		C_10_H_15_NO_2_S	214.09	5.75
	75	Carbamazepine-O-quinone	1135202-29-7	C_15_H_10_N_2_O_3_	289.06	4.77
	76	1,4-Ipomeadiol	53011-73-7	C_9_H_14_O_3_	171.1	6.18
	77	Pyridoxamine 5′-phosphate	529-96-4	C_8_H_13_N_2_O_5_P	249.06	5.63
	78	Obtusilactone A	56522-15-7	C_19_H_32_O_3_	309.24	7.01
	79	2,8-Dihydroxyadenine	30377-37-8	C_5_H_5_N_5_O_2_	185.08	4.24
	80	8-Hydroxy-7-methylguanine	1688-85-3	C_6_H_7_N_5_O_2_	199.09	4.83
	81	2′-Deoxysepiapterin	1797-87-1	C_9_H_11_N_5_O_2_	239.13	6.18
	82	Norfuraneol	19322-27-1	C_5_H_6_O_3_	115.04	5.59
	83	Osmundalactone	69308-39-0	C_6_H_8_O_3_	129.05	5.59
Benzenoids	84	7-Benzylidenenaltrexone		C_27_H_27_NO_4_	428.19	7.36
	85	1,2-Dihydronaphthalene	447-53-0	C_10_H_10_	131.09	6.2
	86	(4-Methylphenyl) acetaldehyde	104-09-6	C_9_H_10_O	135.08	6.2
	87	Dipivefrin	52365-63-6	C_19_H_29_NO_5_	369.24	7.07
	88	Ibopamine	66195-31-1	C_17_H_25_NO_4_	325.21	7.18
Organic oxygen compounds	89	3-Methylcyclopentadecanone	541-91-3	C_16_H_30_O	239.24	7.07
	90	2,3-Butanediol	513-85-9	C_4_H_10_O_2_	91.08	4.77
Amino acids, peptides, and analogs	91	Asp Phe Met Pro		C_23_H_32_N_4_O_7_S	553.2	7.46
	92	Ala Asp Phe Met		C_21_H_30_N_4_O_7_S	527.18	7.36
	93	Asp Gly Asn Arg		C_16_H_28_N_8_O_8_	459.2	7.36
	94	Cys Gly Gly Gly		C_9_H_16_N_4_O_5_S	147.05	4.75
	95	Gly Arg His		C_14_H_24_N_8_O_4_	369.2	7.36
	96	Ala His Arg Arg		C_21_H_38_N_12_O_5_	556.34	6.65
	97	Stearoylglycine	6333-54-6	C_20_H_39_NO_3_	342.3	6.59
	98	Ile Glu Asp		C_15_H_25_N_3_O_8_	393.2	7.18
	99	N-Stearoyl GABA	52558-71-1	C_22_H_43_NO_3_	370.33	6.99
	100	Behenoylglycine	14246-59-4	C_24_H_47_NO_3_	398.36	7.62
Organosulfur compounds	101	1-(Methylthio)propyl propyl disulfide	126876-22-0	C_7_H_16_S_3_	195.03	4.29
	102	Methanethiosulfonate	44059-82-7	CH_4_O_2_S_2_	110.96	0.78
Phenylpropanoids and polyketides	103	b-D-fructosyl-a-D-(6-O-(E))-feruloylglucoside		C_21_H_28_O_12_	471.15	7.38
Hydrocarbons	104	1-Pentene	109-67-1	C_5_H_10_	71.09	6.18
Alkaloids and derivatives	105	Pipequaline	77472-98-1	C_22_H_24_N_2_	339.18	7.44
	106	10-Hydroxycamptothecin	64439-81-2	C_20_H_16_N_2_O_5_	183.06	4.75
	107	(−)-Aspidospermine	466-49-9	C_22_H_30_N_2_O_2_	377.22	7.36
	108	SN-38	86639-52-3	C_22_H_20_N_2_O_5_	197.08	4.75
Others	109	Sodium palmitate	408-35-5	C_16_H_31_NaO_2_	323.22	7.36
	110	1-(4-Fluorophenyl)-3H-[1,3] thiazolo [3,4-a] benzimidazole	136994-91-7	C_15_H_11_FN_2_S	293.05	4.75

**Table 3:** Statistical results of the number of differential compounds.

Comparison Group Information	Different Compounds Number	Up-Regulated Compounds Number	Down-Regulated Compounds Number
MZLM3-H/MZLM3-L	61	16	45
MZLM6-H/MZLM6-L	86	15	71
MZLM9-H/MZLM9-L	111	84	27
TZL-H/TZL-L	91	14	77
HZL-H/HZL-L	48	4	44
SGJF-H/SGJF-L	147	6	141
SGSF-H/SGSF-L	109	1	108
HLZM-H/HLZM-L	144	47	97
SH-H/SH-L	116	5	111
HLQH-H/HLQH-L	76	4	72
HLMX-H/HLMX-L	92	36	56
GY-H/GY-L	206	133	73

## Data Availability

The original contributions presented in the study are included in the article; further inquiries can be directed to the corresponding authors.

## Supplementary Materials

The following supporting information can be downloaded at: https://www.mdpi.com/article/10.3390/foods15010037/s1, Table S1: Identification and quantitation of non-volatile flavor compounds in strong-flavor baijiu. Table S2: Critical metabolites (VIP > 1, *p* < 0.05) responsible for the metabolomics variation between high- and low-alcohol SFB. Table S3: Classification information for samples of different brands and origins.
