# Supplementary material for: Differential Non-Volatile Metabolomics in High- and Low-Alcohol Strong-Flavor Baijiu by Non-Targeted Approach
Source: Foods. 2025 Dec 22;15(1):37. doi: 10.3390/foods15010037 (PMC12785947; doi:10.3390/foods15010037)
Supplement: Supplementary file 1 [file foods-15-00037-s001.zip › Table S3.pdf]

Table S3. Classification information for samples of different brands and origins.

Brands	Samples of High Alcohol	Samples of Low Alcohol	Origins	Samples of High Alcohol	Samples of Low Alcohol
MZL	MZLM3-H	MZLM3-L	YHSQ	MZLM3-H	MZLM3-L
	MZLM6-H	MZLM6-L		MZLM6-H	MZLM6-L
	MZLM9-H	MZLM9-L		MZLM9-H	MZLM9-L
TZL	TZL-H	TZL-L		TZL-H	TZL-L
HZL	HZL-H	HZL-L		HZL-H	HZL-L
SG	SGJF-H	SGJF-L		HLZM-H	HLZM-L
	SGSF-H	SGSF-L		SH-H	SH-L
HLZM	HLZM-H	HLZM-L		HLQH-H	HLQH-L
SH	SH-H	SH-L		HLMX-H	HLMX-L
HLQH	HLQH-H	HLQH-L	SHSQ	SGJF-H	SGJF-L
HLMX	HLMX-H	HLMX-L		SGSF-H	SGSF-L
GY	GY-H	GY-L	LSHA	GY-H	GY-L
